# Paying to participate?

**DOI:** 10.1002/sctm.21-0015

**Published:** 2021-05-19

**Authors:** Nancy M.P. King

**Affiliations:** ^1^ Department of Social Sciences and Health Policy and Wake Forest Institute for Regenerative Medicine Wake Forest School of Medicine, and Bioethics Graduate Program and Center for Bioethics, Health, and Society, Wake Forest University Winston‐Salem North Carolina USA

**The question:** To participate in a clinical trial, is it okay to pay?

**The answer:** It depends.

## 1 A LITTLE HISTORY

In the good old days of apocryphal history, people who enrolled in clinical trials had one altruistic goal: to contribute to generalizable knowledge. To facilitate this generous partnership for the good of science and to acknowledge the difference between helping to test the safety and efficacy of unproven interventions and receiving standard treatments, participants were not charged for any aspect of their participation. They received diagnostic tests and experimental interventions for free and were often reimbursed for incidental costs, such as mileage and parking. The resulting apparently clear separation between research and treatment was by no means as distinct as imagined, however, because patients with the disease or condition being studied, as well as healthy people who lacked adequate health insurance, often enrolled in research in order to receive standard and experimental diagnostic and treatment interventions to which they would otherwise have no access. The increasingly unclear line between research and treatment was publicly and definitively blurred during the AIDS epidemic, when “research is our treatment” became the mantra of HIV+ patients who sought access to unproven medications in the absence of effective treatments and in the face of otherwise certain death.^1^

The outcome of AIDS activism was effective reform of the clinical trial process. Over time, access to trial participation was increased, research design became more flexible, trial conduct was speeded up, and alternative access pathways were created.^2^ During the same time period, public expectations about the capacity of science to develop curative treatments also increased greatly. Skepticism grew about the need for the still‐careful and deliberate process of evidence‐gathering upon which biomedical science necessarily relies, and many patients understandably wanted science to move even faster on their behalf.

The moral responsibilities of scientists include the duty to make only fair and reasonable offers of research enrollment to potential participants; thus, research reforms continue to protect the integrity of the process of evidence‐gathering, in order to help ensure that only safe and effective interventions are approved as treatments for future patients.^3^, ^4^ Nonetheless, patients with dire diagnoses may well argue for the right to take excessive risks in order to try unproven interventions. This perspective led to the controversial “right to try” movement, which enables some patients to obtain early access to unproven interventions without enrolling in clinical trials, as long as they are willing and able to pay for the intervention and accompanying costs.^5^

At the same time, the costs of providing research interventions without charge to participants continue to escalate. Thus, academic medical centers began to ask health insurers to reimburse researchers for diagnostic and therapeutic interventions that would ordinarily be paid for by health insurance if provided to patients outside the research context. As a result, patients enrolled in research studies face new and unexpected costs for research participation, in the form of health insurance deductibles and copayments. These costs can be considerable, especially for cancer patients who participate successively in studies of new interventions after standard treatments have failed them. This pattern of research participation is common, and may prevent or slow disease progression for some patients, but it can be financially ruinous.^6^ Indeed, this partial payment requirement may make some researchers reluctant to recruit uninsured and underinsured patients as participants. When neither researchers nor their medical centers can afford to provide standard diagnostic and treatment interventions at no cost in clinical trials, patient‐participants who lack adequate health insurance would face large bills if they enrolled and were then charged as they would be when seeking treatment.

Against this backdrop, the development of pay‐to‐participate trials has also influenced clinical research. At first, when researchers asked potential participants to pay in order to enroll in their studies, it seemed clear that the trials in question had failed to secure funding, or avoided trying, because their science was shaky. Scandalous examples of trials charging enormous sums were reported by medical journalists^7^, ^8^; medical centers and their institutional review boards (IRBs) prevented researchers from billing their subjects; and everyone believed that the problem was solved until some clinics, capitalizing on public confusion about stem cells, began to charge patients for receiving unproven interventions, sometimes labeling the charges as research participation costs and sometimes calling them charges for treatment.^9^

Yet something else is happening that has muddied these waters even more: federal funding for biomedical research has been shrinking, but the number of researchers seeking funding support continues to grow. Thus it is no longer easy to say that every study failing to secure funding represents poor science; instead, some might have barely missed the funding cutoff, and could have received funding a year or two earlier. How is the public to know whether a pay‐to‐participate trial represents bogus science or a potential breakthrough that the study section was unwilling to take a chance on?

## 2 FEDERAL RECOMMENDATIONS

For this reason, in late 2019 the DHHS Secretary's Advisory Committee on Human Research Protections (SACHRP) submitted recommendations on charging subjects for clinical trial participation to Secretary Alex Azar.^10^ These recommendations take the form of a “points to consider” document examining the ethical and regulatory issues that IRBs and potential research participants should consider when determining whether charging subjects for participation in a particular trial is acceptable. These recommendations have not been adopted as or adapted into guidance by the Office for Human Research Protections. However, all SACHRP recommendations are publicly available^11^ and frequently consulted by IRBs.

In this article, “SACHRP acknowledges that there may be legitimate reasons to charge research subjects, and that prohibiting pay‐to‐participate trials could in some instances preclude valuable research.” Beginning “from the position that whenever possible, it is best to avoid charging for research participation,” the Committee identifies “considerations that can be used to balance the benefits and harms of this research funding model.”^12^ The document lists reasons why sponsors might seek payment from subjects, including lack of sufficient federal funding to support all worthwhile projects, development costs so significant as to make charging for an investigational product necessary in order to make conducting a clinical trial feasible, trial sponsorship by patient advocacy groups with limited access to conventional funding sources, and finally profit‐seeking, that is, using the pay‐to‐participate research model as a way to avoid the prohibition against marketing investigational products.

SACHRP notes that the FDA permits charging for investigational drugs and devices when the charges do not exceed the sponsor's production costs and when those costs would otherwise make it impossible to develop a promising intervention. A compelling justification is required.^13^

Potential participants' reasons for willingness to pay are of course most likely to be their hopes of direct medical benefit from the investigational product. Well‐informed potential subjects can make reasonable choices if they recognize that benefit is uncertain; still, paying for the intervention may strengthen participants' certainty that they will benefit, thus greatly increasing the likelihood of therapeutic misconception.

In light of these considerations, SACHRP's recommendations to IRBs and potential participants take the form of questions that should be asked and thoroughly examined. IRBs need to determine the following:

- Does the study meet relevant thresholds of scientific quality?
- Is the harm‐benefit balance justifiable?
- Why is traditional funding not being used, and is there any way other than charging subjects to fund the study?
- Will charging subjects unfairly limit who can enroll, or make it more likely that subjects will consider the unproven intervention to be an effective treatment? If so, are there ways to mitigate these concerns?

SACHRP then suggests numerous ways for IRBs and investigators to reduce the risks posed by a pay‐to‐participate trial that is scientifically problematic or misleading. Remedies range from refusing to approve a protocol without additional scientific review showing validity and value to strengthening the consent form and process.^14^

The recommendations conclude with an extensive list of questions that patients should ask when they consider joining a pay‐to‐participate trial. This list of questions is intended to remind potential participants to expect detailed and meaningful information about particular studies and to facilitate reasoned decision‐making about their participation. The list of questions includes^15^:

- Who has reviewed this research and said it is OK to do? Has there been ethical and scientific review of the research?
- Why do the researchers think that what is being tested might help people like me? What do they think it might do, if it works?
- Why am I being asked to pay to join this research?
- How much will the researchers charge me to be in this research?
- What exactly will I be paying for (eg, diagnostic tests, the study intervention)?
- If I quit the research, will I get any of my money back?
- If the researchers make me leave the research, will I get any of my money back?

## 3 CONCLUSION

Paying to participate is an atypical but potentially acceptable means of facilitating valuable research that could not otherwise go forward. However, IRBs and potential participants should require investigators to communicate thoroughly and meaningfully and to provide compelling reasons for using this funding method. Without good‐faith efforts by investigators, sponsors, and oversight bodies, the risk that desperate patients will view paying for research as their only hope is too great. However, transparency can help build trustworthiness and set the stage for reasoned decision‐making in an increasingly complex research environment.^16^

## CONFLICT OF INTEREST

The author indicated no potential conflicts of interest.
